# Combination of CRP and miRNA signature as a potential diagnostic strategy for Kawasaki disease

**DOI:** 10.3389/fped.2025.1678095

**Published:** 2025-10-15

**Authors:** Xiaoyan Huang, Huiting Li, Xiangrong Zhao, Haixiang Zhang, Yaping Li, Qian Niu, Jiaojiao Wang, Cuixiang Xu

**Affiliations:** ^1^ Shaanxi Provincial Key Laboratory of Infection and Immune Diseases, Shaanxi Provincial People’s Hospital, Xi’an, Shaanxi, China; ^2^ Shaanxi Engineering Research Center of Cell Immunology, Shaanxi Provincial People’s Hospital, Xi’an, Shaanxi, China; ^3^ Department of Pediatrics, Shaanxi Provincial People’s Hospital, Xi’an, Shaanxi, China; ^4^ Department of Graduate School, Yan’an University, Yan’an, Shaanxi, China

**Keywords:** Kawasaki disease, biomarker, C-reactive protein, microRNA, diagnostic strategy

## Abstract

**Background:**

Kawasaki disease is the leading cause of acquired heart disease in children, yet timely diagnosis remains difficult due to overlapping symptoms with other febrile illnesses.

**Methods:**

In a retrospective case–control study of 38 children with Kawasaki disease and 44 febrile controls, we measured hematological parameters and C-reactive protein (CRP) using standardized analyzers and profiled seven serum microRNAs by qRT-PCR. Biomarkers showing significant differences were used to build logistic regression models with a 70/30 train–test split, and diagnostic accuracy was assessed by receiver operating characteristic analysis. Functional enrichment of miRNA targets was explored using network analysis.

**Results:**

CRP and three microRNAs (miR-223-3p, miR-19a-3p, miR-18a-5p) were significantly elevated in Kawasaki disease. Individually, these markers achieved strong discrimination (AUC: 0.846–0.986), while their combination yielded an AUC of 0.990, sensitivity 1.000, and specificity 0.923. The three microRNAs were positively correlated and enriched for pathways including p53 signaling and cell cycle regulation, with KCNQ1OT1 identified as a shared lncRNA interactor.

**Conclusion:**

Integrating CRP with a concise serum miRNA panel demonstrates promising discriminatory potential for Kawasaki disease vs. other febrile illnesses and suggests mechanistic involvement of p53-associated pathways, supporting future validation in larger, independent cohorts.

## Introduction

Kawasaki Disease (KD), clinically referred to as mucocutaneous lymph node syndrome, is an acute febrile illness primarily affecting children under the age of 5 rears, with etiology yet to be fully understood (1). It is the leading cause of acquired heart disease in children, with approximately 25% of untreated cases developing coronary artery lesions (CAL), potentially leading to myocardial infarction or sudden death (2). KD affects numerous ethnic groups globally, with East Asian countries having the highest incidence, notably China (3). Currently, KD diagnosis relies heavily on clinical features, with no confirmatory laboratory test available to date (4). However, resembling clinical features of KD are also presenting in many other childhood infectious febrile illnesses (5, 6), such as viral infections and scarlet fever (7). Consequently, discriminating KD from these conditions can pose a significant diagnostic challenge. Misdiagnosis can result in treatment delays, substantially elevating the risk of developing CAL and associated morbidity and mortality. Early intravenous immunoglobulin (IVIG) administration can reduce CAL risk to 5% (8). Therefore, there is an urgent demand for developing a more specific, sensitive, accurate, reproducible, and rapid biochemical test to facilitate a definitive diagnosis and timely initiation of appropriate therapeutic interventions.

MicroRNAs (miRNAs) are small non-coding RNA molecules, usually 18–25 nucleotides in length that play a critical role in regulating cellular and immune functions through post-transcriptional modifications (9). MiRNAs are implicated in various pathological abnormalities, ranging from cancer (10) to adult and pediatric cardiovascular diseases (11–13). Specific to KD, miRNAs have emerged as potential diagnostic biomarkers and critical players in disease pathogenesis. Initial studies by Shimizu et al. highlighted the differential expression of miRNAs in acute KD, notably miR-145, which influences TGF-β signaling in coronary arteries (14). Subsequent research has expanded our understanding of miRNAs in KD diagnosis, pathogenesis, and treatment (15). For instance, Yun et al. found that miR-200c and miR-371-5p upregulation could be implicated in KD pathogenesis by modulating the inflammatory response (16). John et al. reported a distinctive circulating miRNA profile in KD, including miR-210-3p, miR-184, and miR-19a-3p, which are positively correlated. Moreover, Jia et al. characterized a set of four serum exosomal miRNAs, miR1246, miR-4436b-5p, miR-197-3p, and miR-671-5p, that are capable of differentiating KD patients from healthy individuals (17). Furthermore, bioinformatic analysis has led to specific microRNAs such as mir-126-3p, mir-375, and mir-146a-5p being identified as potential biomarkers for KD (18). Recent investigations have identified ten miRNAs holding substantial promise as valuable biomarkers for KD and may participate in KD pathogenesis via regulation of the TGF-β signaling pathway (19). Despite significant progress, a universally accepted miRNA-based diagnostic approach for KD has yet to be established in clinical practice, reflecting the ongoing need for research in this area.

In this study, we aimed to assess the efficacy of a novel multi-biomarker test in distinguishing KD from non-KD febrile controls. Our focus was on a combination of conventional and novel biomarkers: two widely recognized inflammatory markers, C-reactive protein (CRP) and platelet count (PLT), along with seven miRNAs (miR-223-3p, miR-19a-3p, miR-18a-5p, miR-21-5p, miR-155-5p, miR-145, and miR-146a-5p) identified for their involvement in KD. CRP and PLT are established nonspecific indicators of inflammation and infection (20, 21), with documented elevations in acute KD cases (22, 23). However, while CRP demonstrates high sensitivity, its limited disease specificity and frequent elevation in various viral or bacterial infections reduce its standalone diagnostic utility. PLT reflects systemic inflammation and thrombocytosis, commonly observed in KD after day 5, but also lacks disease specificity. In contrast, circulating miRNAs provide molecular-level insight into disease-specific immunopathological responses and may complement conventional markers by capturing regulatory signatures unique to KD. Therefore, we hypothesized that integrating CRP and PLT with selected miRNAs could enhance diagnostic accuracy by leveraging both systemic inflammatory markers and post-transcriptional regulatory signals. This approach aims to improve diagnostic precision in real-world cases where clinical presentations often overlap between KD and other febrile illnesses. Among the seven selected candidate miRNAs, miR-223-3p (24), miR-19a-3p (25), miR-18a-5p (26), miR-145 exhibit an elevated pattern in KD, while miR-21-5p and miR-155-5p were shown to be downregulated (27). MiR-146a-5p has been pinpointed as a direct target of differentially expressed genes (DEGs) in KD through network analysis, and *miR-146a* gene polymorphisms have been associated with KD susceptibility (28).

We conducted a retrospective case–control analysis involving 38 blood samples from patients diagnosed with KD and 44 samples from febrile control children to evaluate the expression levels of CRP, PLT, and selected serum miRNAs. Beyond confirming the individual discriminatory value of each biomarker, we demonstrated that their combined application significantly enhances diagnostic performance. Additionally, we investigated the interrelationships among these biomarkers and performed functional enrichment analyses to explore their involvement in molecular pathways relevant to KD pathophysiology, aiming to provide further insight into their clinical utility and mechanistic significance.

## Materials and methods

### Patients and clinical data

We retrospectively analyzed the medical records of children admitted to Shaanxi Provincial People's Hospital (China) between June 2022 and June 2024 with a diagnosis of Kawasaki disease (KD) or other febrile illnesses. A total of 42 children with KD and 46 with febrile illnesses were initially considered. However, 4 KD patients and 2 febrile patients were excluded due to withdrawal from the study. The final cohort included 38 KD patients (16 with complete KD and 22 with incomplete KD) and 44 febrile controls. All patients in the KD group met the diagnostic criteria established by the American Heart Association for complete or incomplete KD.

The children in the non-KD febrile control cohort have been diagnosed with a range of illnesses, such as infantile exanthem (6 cases), urticaria (7 cases), acute pharyngitis (5 cases), acute purulent tonsillitis (4 cases), conjunctivitis (5 cases), sepsis (3 cases), various viral (including enterovirus, 6 cases) and bacterial infections (8 cases). During this time, all of the individuals' age, sex, days of fever duration, Hemoglobin, Red blood cell count (RBC), White blood cell count (WBC), Neutrophil count, PLT and CRP were gathered.

The Shaanxi Provincial People's Hospital Ethics Committee evaluated and approved this study (No. 2022114). Prior to sample collection, the patient's parents or legal guardians provided written informed consent. Every experiment was carried out in accordance with the rules and regulations that were in effect at the time.

The following criteria were established for the KD cohort's inclusion:

I. The patient meets the diagnostic criteria for KD, as defined by the American Heart Association (28). This includes having a fever that is higher than 38.0°C and lasts for more than five days. Additionally, the patient must exhibit at least two of the following clinical features: polymorphous rash, bilateral conjunctivitis, cervical lymphadenopathy, diffuse oral mucosal congestion, and edema or erythema of the hands or feet;
II. First-time incidence of the disease;
III. Age under 14;
IV. Full clinical data;
V. Children's guardians have informed consent to this study.

The following were established as the KD cohort's exclusion criteria:

I. Pre-admission treatment with immunoglobulin, aspirin, or other medications;
II. Pre-existing conditions such as congenital heart disease, myocarditis, arrhythmias, and systemic diseases affecting organs like the liver, kidneys, lungs, and malignancies, hematological disorders, and genetic metabolic diseases;
III. Accompanied with autoimmune diseases;
IV. Subjects who withdrew midway.

### Sample collection

Peripheral venous blood (PVB) samples were collected from KD and febrile control patients within 24 h of inpatient admission (i.e., on illness days 5–7), and prior to treatment with intravenous immunoglobulin, aspirin, or other disease-specific interventions. PLT, hemoglobin, RBC, WBC and neutrophils were measured by the Sysmex XN-2000 Hematology Analyzer using the impedance flow cytometry method (Sysmex et al., Japan). CRP levels were assessed using an automatic specific protein analyzer using the nephelometric method (PA-990; Lifotronic, Shenzhen, China). All procedures followed the operating protocols and Standard Operating Procedures (SOP) documentation of the reagent and instrument manufacturers.

### miRNA isolation and qRT-PCR

Total RNA from serum samples was extracted with the RNeasy plus mini kit (Qiagen), and the RNA concentration was quantified using a Nanodrop 3000 Spectrophotometer (Thermo et al., USA). MiRNAs were reverse transcribed to cDNA with the PrimeScript RT reagent kit (TaKaRa, Dalian, China), and then detected using specific primers synthesized by Sangon Biotech (Shanghai, China) in an ABI 7500 Real-Time PCR System 3 (Applied Biosystems, Foster City, CA), with U6 being the internal control. The expression of miRNAs was calculated and represented using the 2^−*ΔΔ*CT^ method. Quantitative RT-PCR primer sequences were in Table 1.

**Table 1:** Primer sequences of real-time PCR.

Gene	Primer sequences
Human-miR-223-3p	F: 5′-TCCCAACTGGCTCAAGTTCC-3′
Human-miR-18a-5p	F: 5′-GCTAAGGTGCATCTAGTGCAGATAG-3′
Human-miR-21-5p	F: 5′-TAGCTTATCAGACTGATGTTGA-3′
Human-miR-19a-3p	F: 5′-TGAGCAAAACAAGCAAATCTGA-3′
Human-miR-145	F: 5′-CAGTGCGTGTCGTGGAGT-3′
Human-miR-146a-5p	F:5′-ACACTCCAGCTGGGTGAGAACTGAATTCCA-3′
Human-U6	F: 5′-CTCGCTTCGGCAGCACA-3′

### Construction of miRNA interaction network and functional analysis

miRNet 2.0 tool was used to map the documented interactions between miRNAs and target genes or lncRNAs, and to perform functional enrichment analysis (29). miRNet 2.0 is an interactive platform integrating major databases to elucidate and construct networks between and among experimentally validated target genes, proteins, lncRNAs, circRNAs, and sncRNAs. In miRNet 2.0, the human peripheral blood-specific miRNA-gene interaction data was extracted from latest miRNA annotation databases including miR Base (30), miTarBase (31), DIANA-TarBase (32), TSmiR (33) and IMOTA (34). The miRNA-lncRNA interactions were collected from starBase (35). Functional enrichment analysis was performed using GO and KEGG pathways, implementing hypergeometric tests and empirical sampling enrichment algorithms. The visual analytics was enhanced by methods including the prize-colleting Steiner Forest (PCSF) algorithm. The backbone layout was used in visual representation to effectively reveal hidden patterns in large networks and emphasize the most embedded edges (36). It was manually built by Cytoscape.

### Statistical analysis

Data collected for demographic and clinical characteristics was assessed for Gaussian distribution with the Shapiro–Wilk test. Variations in age, PLT and all miRNAs among KD patients and other febrile control cohorts were presented as median values and interquartile ranges (IQR), and the intergroup comparisons were performed using Mann–Whitney *U* test. Variations in days of fever duration, hemoglobin (g/L), RBC count, WBC count (×10^9^/L), neutrophil count (×10^9^/L) and CRP were presented as mean ± SD, and the intergroup comparisons were performed using unpaired *t*-test. Variations in sex were presented as frequency (*n*) and percentage (%), and the intergroup comparisons were performed using Fisher's exact test. To account for multiple comparisons and control the family-wise error rate, the Holm-Šídák correction was applied to all *p*-values obtained from the statistical tests. This method was chosen due to its balance between controlling Type I error and maintaining statistical power, especially in the context of multiple hypothesis testing involving the evaluation of several biomarkers. *P*-values were ranked, and adjusted significance levels were calculated accordingly for each test. Adjusted *p*-values are reported throughout the manuscript, and updated figures reflect these adjustments. Outliers were winsorized at the values of three times the median absolute deviation or greater than the upper limit of quantification. Only variables that demonstrated statistical significance were recruited in the following assessments.

Multiple logistic regression with backward selection was used to assess the predictive value of the candidate biomarkers (individual and combined) for the differentiation of KD *vs.* other febrile diseases. Data were randomly split into training and test sets to prevent overfitting and to validate the diagnostic models. The training set comprised 70% of the total dataset, while the remaining 30% constituted the test set. This split was performed to ensure that the diagnostic models were trained and tested on independent samples. The model was trained using the training dataset, optimizing for maximum Youden's J statistic to identify the optimal cut-off points for each biomarker. The trained logistic regression models were then validated using the independent test set. Receiver operating characteristic (ROC) curves were generated for each biomarker and the combined model using the test set to evaluate their diagnostic performance. The area under the ROC curve (AUC) was calculated to assess the overall ability of the biomarkers to differentiate between KD and controls. The optimal cut-off values, sensitivity, specificity, and maximum Youden's J statistics were also calculated to determine the best threshold for KD diagnosis. Confidence intervals for AUCs were computed using DeLong's method to assess the statistical significance of the diagnostic performance.

Pearson's correlation coefficient was applied to determine the relationships between candidate biomarkers. A *p* < 0.05 was considered to be statistically significant.

GraphPad Prism version 10.0 (GraphPad Software, San Diego, California, USA) and RStudio Team (RStudio: Integrated Development for R. RStudio, PBC, Boston, MA URL http://www.rstudio.com/, 2020) was used for all statistical analysis. The pROC package was used for ROC analysis.

## Results

### Patient characteristics

Demographic and clinical characteristics were recorded and analyzed for the KD patients and febrile controls enrolled in this study. The baseline characteristics, containing Age, Sex, days of fever duration, Hemoglobin, RBC, WBC, Neutrophil count, and CRP were not significantly different between the KD and non-KD febrile cohorts, but a significant difference was shown in CRP between the two cohorts, with KD patients demonstrating a higher level of CRP (Table 2).

**Table 2:** Demographic and clinical characteristics of KD patients and non-KD febrile controls cohorts.

Characteristics	KD patients (*n* = 38)	Non-KD Febrile controls (*n* = 44)	*p* value
Age (y)	3.3 (1.0–5.2)^a^	4.6 (2.0–7.1)^a^	0.148^c^
Sex (female, %)	18 (47.37%)	23 (52.27%)	0.825^d^
days of fever duration	5.46 ± 1.04^b^	5.18 ± 2.19^b^	0.786^e^
Hemoglobin (g/L)	110 ± 14.57^b^	104 ± 12.88^b^	0.774^e^
RBC count (x10^12^/L)	4.23 ± 0.57^b^	4.50 ± 0.94^b^	0.153^e^
WBC count (x10^9^/L)	18.55 ± 2.07^b^	22.08 ± 2.59^b^	0.407^e^
Neutrophil count (x10^9^/L)	12.68 ± 1.64^b^	15.18 ± 9.87^b^	0.361^e^
PLT (x10^9^/L)	486.0 (407.0–662.0)^a^	537.0 (400.0–655.3)^a^	0.7972^c^
CRP (mg/L)	98.45 ± 46.09^b^	39.05 ± 23.58^b^	<0.0001^e^

Data was presented as ^a^median, IQR or ^b^mean ± SD; *p* values were calculated by ^c^Mann–Whitney test; ^d^Fisher's exact test; ^e^unpaired *t* test.

KD, Kawasaki disease; RBC, red blood cell count; WBC, white blood cell count; PLT, platelet count; CRP, C-reactive protein.

### CRP and miRNAs differentiate patients with KD from those with non-KD febrile diseases

**Figure 1:**
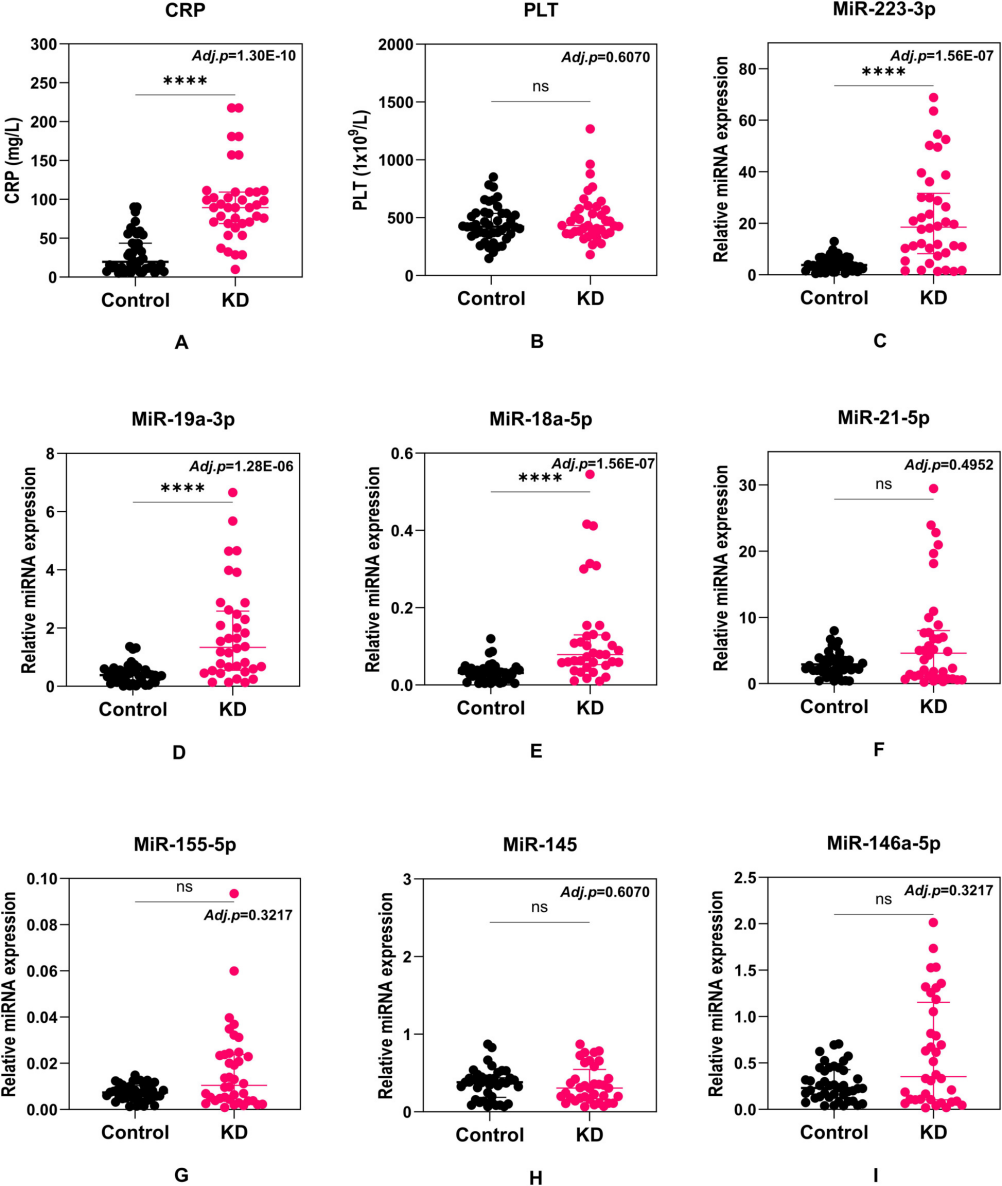
Comparison of C-reactive protein (CRP) **(A)**, platelet count (PLT) **(B)**, miR-223-3p **(C)**, miR-19a-3p **(D)**, miR-18a-5p **(E)**, miR-21-5p **(F)**, miR-155-5p **(G)**, miR-145 **(H)**, and miR-146a-5p **(I)** levels between KD and non-KD febrile cohorts. Each dot represents a unique patient. Median values and interquartile ranges are shown in the figures. Statistical differences were calculated with the Mann–Whitney *U* test. Adjusted *p* values were calculated using the Holm-Šídák test and shown on the figures.

First, we aimed to determine the differences between these selected candidate biomarkers in KD patients and non-KD febrile control cohorts. Seven candidate miRNAs were evaluated for their relative expression using the qRT-PCR method. A total of nine candidate biomarkers (including PLT and CRP) were analyzed to test statistical significance between the two cohorts (Figure 1). For all analyses involving multiple comparisons, the *p*-values were adjusted using the Holm-Šídák correction to control for multiple testing. The revised *p*-values are reported in the corresponding sub-figures. Among these biomarkers, 4 out of 9 demonstrate significant differences, including one conventional protein biomarker (CRP) (Figure 1A) and three miRNAs (miR-223-3p, miR-19a-3p and miR-18a-5p) (Figures 1C–E), of which an elevated pattern was demonstrated in KD patients compared to patients with other febrile diseases. These four potential biomarkers were then recruited in the following assessments. No statistical difference was found in PLT levels (Figure 1B) or relative expression of miR-21-5p, miR-155-5p, miR-145, or miR-146a-5p (Figures 1F–I).

**Figure 2:**
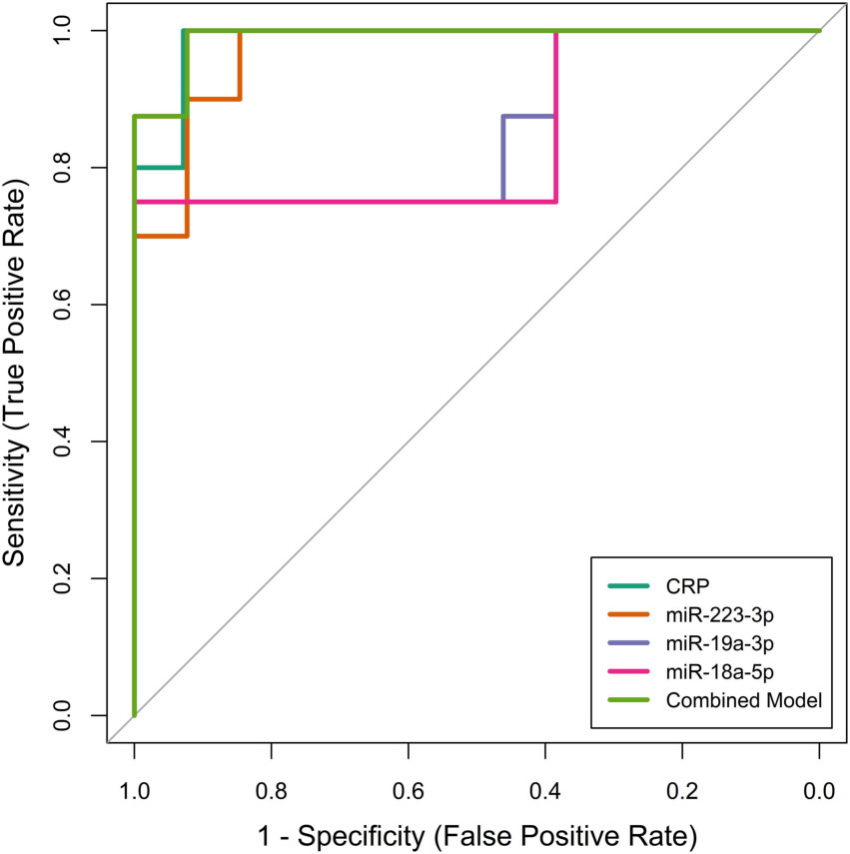
Receiver operating characteristic (ROC) curves illustrating the diagnostic performance of individual biomarkers (CRP, miR-223-3p, miR-19a-3p, miR-18a-5p) and their combined model in differentiating patients with kawasaki disease (KD) from those with non-KD febrile diseases. The ROC curves were generated using an independent test set to validate the diagnostic accuracy of each biomarker and the combined model.

We then evaluated the performance of these four selected potential biomarkers in discriminating patients with KD from those with non-KD febrile diseases. Receiver operator characteristic (ROC) curves were plotted for each biomarker (Figure 2). By training the diagnostic models on a separate dataset and validating them with an independent test set, we ensured robust evaluation of the biomarkers' diagnostic capabilities, mitigating the risk of overfitting and providing more realistic estimates of model performance. The ROC analysis demonstrated that all biomarkers, including CRP, miR-223-3p, miR-19a-3p, miR-18a-5p, and their combined model, had significant diagnostic ability in differentiating KD from non-KD febrile controls (Table 3). CRP exhibited the highest individual diagnostic performance with an AUC of 0.986 (95% CI: 0.952–1.000), a sensitivity of 1.000, a specificity of 0.929, and an optimal cut-off value of 74.97 mg/L. MiR-223-3p followed with an AUC of 0.969 (95% CI: 0.912–1.000), a sensitivity of 1.000, and a specificity of 0.846. The optimal cut-off value for miR-223-3p was 8.356. MiR-19a-3p and miR-18a-5p displayed moderate diagnostic capabilities with AUCs of 0.856 (95% CI: 0.658–1.000) and 0.846 (95% CI: 0.637–1.000), respectively. Both had sensitivities of 0.750 and specificities of 1.000 at cut-off values of 1.105 and 0.047, respectively. The combined model of all biomarkers showed the highest overall diagnostic performance with an AUC of 0.990 (95% CI: 0.964–1.000), sensitivity of 1.000, specificity of 0.923, and an optimal cut-off value of 0.705. This exploratory diagnostic approach, integrating conventional biomarkers with miRNA profiling, demonstrates promising discriminatory potential in differentiating KD from other febrile illnesses. While the model's performance was high in internal validation, its application remains preliminary due to the limited sample size and warrants further evaluation in larger, independent cohorts.

**Table 3:** Performance metrics for differentiating biomarkers' ROCs.

Biomarkers	AUC	95% CI	Max. J	Sensitivity	Specificity	Cut-off value
CRP	0.986	0.952–1.000	1.929	1.000	0.929	74.97 (mg/L)
miR-223–3p	0.969	0.912–1.000	1.846	1.000	0.846	8.356
miR-19a-3p	0.856	0.658–1.000	1.750	0.750	1.000	1.105
miR-18a-5p	0.846	0.637–1.000	1.750	0.750	1.000	0.047
Combined	0.990	0.964–1.000	1.923	1.000	0.923	0.705

The corresponding areas under the ROC curve (AUCs) of four individual or combined biomarkers are listed within the columns. The optimal cut-off value was calculated from Youden's J statistic. 95% CI, sensitivity and specificity, and *p* values were reported.

CI, confidence interval; Max. J, maximum Youden's J statistic.

**Figure 3:**
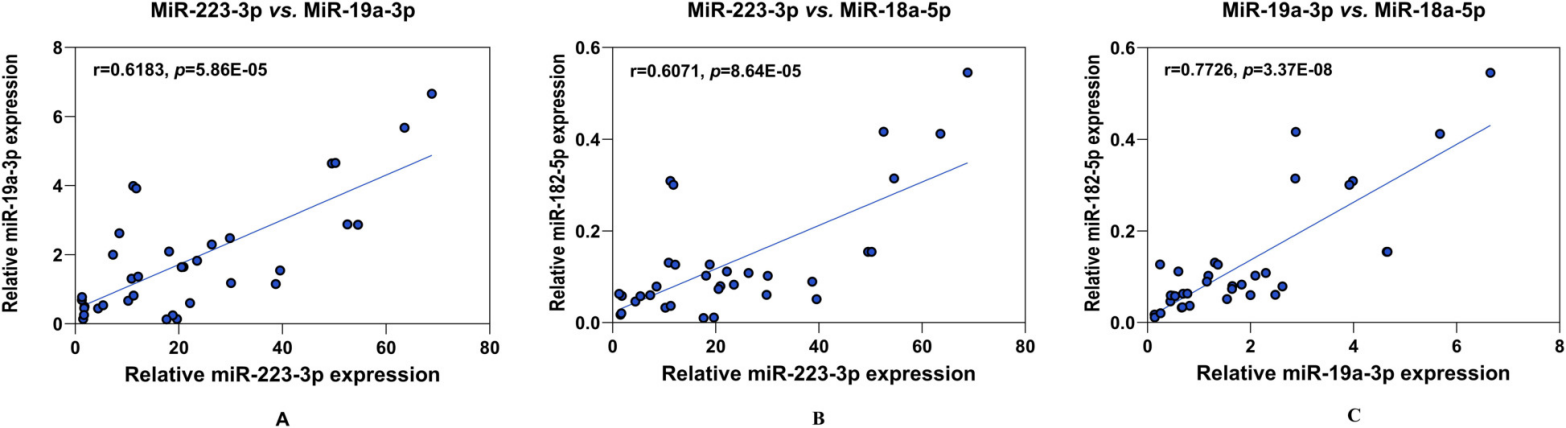
Analysis of the correlation between the four potential biomarkers of KD. The positive paired correlation of the relative expression is shown between miR-223-3p and miR-19a-3p **(A)**; miR-223-3p and miR-18a-5p **(B)**; and miR-19a-3p and miR-18a-5p **(C)**, respectively, using Pearson's coefficient analysis (all, *p* < 0.05). CRP is not corelated with any miRNAs (data not shown). r, Spearman's rank correlation coefficient.

We then examined the potential relationships between these potential biomarkers using Pearson's coefficient analysis. Notably, the relative expression of miR-223-3p, miR-19a-3p, and miR-18a-5p were highly consistent and positively correlated (all *p* < 0.05) (Figure 3).

### MiRNA interaction network and functional analysis

To investigate the functions of the identified candidate miRNAs (miR-223-3p, miR-19a-3p and miR-18a-5p) shown to be discriminative of KD and other febrile diseases and how these functions are implicated in the development and progression of KD, we first utilized the miRnet 2.0 tool to obtain downstream gene targets of these miRNAs. The human peripheral blood-specific miRNA-gene interaction data was extracted from central databases, including miTarBase v8.0, DIANA-TarBase v8.0, TSmiR, and IMOTA.

**Figure 4:**
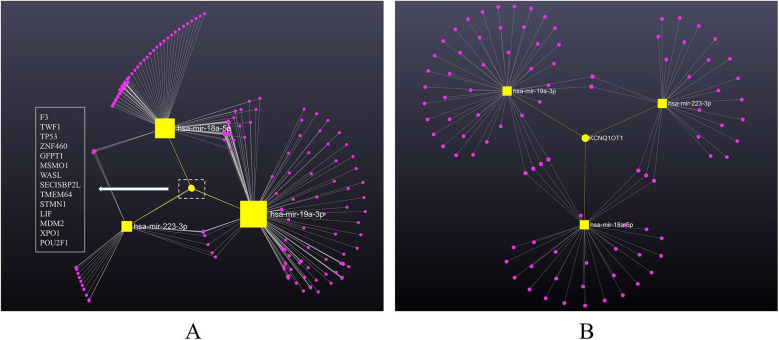
MiRNA-gene/lncRNA interaction network showing the genes associated with three miRNAs, constructed using cytospace. MiRNAs are labeled and shown in yellow squares sized by the number of interactions. **(A)** MiRNA-gene network. The target genes are shown in pink dots. Edges between nodes indicate miRNA-gene interaction. The yellow dot represents the identified 14 commonly shared gene targets of the miRNAs, listed in the left panel. **(B)** MiRNA-lncRNA network. The target lncRNAs are shown in pink dots. Edges between nodes indicate miRNA-lncRNA interaction. The yellow dot represents the identified commonly shared lncRNA target (LncRNA KCNQ1OT1) of the miRNAs.

A total of 2,714 gene targets was identified to be interacting with all three miRNAs (Supplementary Table S1), among which 14 were commonly shared, being F3, TWF1, TP53, ZNF460, GFPT1, MSMO1, WASL, SECISBP2l, TMEM64, STMN1, LIF, MDM2, XPO1 and POU2F1 (Figure 4A). These gene targets were validated from multiple methods such as CLASH, next-generation sequencing, PAR-CLIP, luciferase reporter assay and qRT-PCR.

To expand the network further, we integrated starBase to include lncRNAs. According to miRnet 2.0, 94 lncRNAs was identified as directly linked to the three miRNAs (Supplementary Table S2). Intriguingly, lncRNA KCNQ1OT1 was the only lncRNA highlighted to be commonly shared by all three miRNAs (Figure 4B), indicating a potential signature in KD worth exploring. The interactions between miRNAs and target genes/lncRNAs were plotted in the backbone layout using Cytospace software.

To further explore the roles of the identified miRNA-gene network in KD pathophysiology and better understand how this crosstalk contributes to altering specific pathways, we performed functional enrichment analysis in miRnet 2.0 on all the miRNAs and associated genes using KEGG pathways. A total of 100 pathways were identified to be implicated (Supplementary Table S3), among which 32 pathways encompass all three miRNAs (Supplementary Table S4), including “bladder cancer,” “glioma,” “p53 signaling pathway”, “melanoma,” “chronic myeloid leukemia,” “prostate cancer,” “Epstein–Barr virus infection,” “cell cycle” and “steroid biosynthesis.” Considering the functions of interest for KD, a *p*-value < 0.05 and total hits in the gene targets, the “p53 signaling pathway” (*p* = 0.00701) and “cell cycle” (*p* = 0.0222) demonstrated the highest potential to be associated with all three candidate miRNAs and hence in this study, the discrimination between KD and other febrile diseases.

## Discussion

To date, a rapid and effective test for accurate KD diagnosis is still unavailable. This gap complicates the differentiation of KD from other febrile diseases or infections, often leading to treatment delays and the risk of developing coronary artery lesions (CALs). Over the years, different approaches have been employed in the search for potential biomarkers other than routinely used clinical parameters, including but not limited to DNA methylation profiling, particularly detecting HAMP promoter hypomethylation (37), quantitative protein array for cytokines and chemokines, and plasma antibody profiling using proteome microarrays. Cross-sectional studies also identify a broad panel of biomarkers, such as myeloid-related protein 8/14 and the CLEC4D, GPR84, and HP genes in peripheral leukocytes (38). However, a definitive, universally accepted biomarker for KD remains unidentified.

In our study, we evaluated both conventional biomarkers (CRP, PLT) and a novel serum miRNA panel (miR-223-3p, miR-19a-3p, miR-18a-5p, miR-21-5p, miR-155-5p, miR-145, and miR-146a-5p) to assess their diagnostic potential for distinguishing KD from other febrile illnesses (Figure 1). Our primary finding suggests that a multimarker approach—integrating CRP with miR-223-3p, miR-19a-3p, and miR-18a-5p—achieves high discriminative accuracy (AUROC = 0.990, sensitivity = 1.000, specificity = 0.923) (Figure 2). While CRP alone shows relatively high diagnostic utility, its limited disease specificity and overlap with other inflammatory febrile conditions—including viral or bacterial infections—reduce its reliability as a standalone diagnostic marker. Meanwhile, miRNAs offer molecular-level signatures that can capture disease-specific immune regulation patterns not reflected by conventional biomarkers. Therefore, integrating CRP with select miRNAs likely captures both general inflammatory burden and disease-specific regulatory signals. This combined approach may improve diagnostic confidence, particularly in clinically ambiguous cases where conventional parameters alone are inconclusive. It is also important to note that, while the model yielded a sensitivity of 1.000 and specificity of 0.923 in our internal validation cohort, these results must be interpreted with caution due to a relatively limited sample size and potential for overfitting. Nonetheless, this analysis serves as an important proof-of-concept, illustrating the feasibility and potential utility of integrating clinical and molecular biomarkers for improving KD diagnostic specificity. Future multicenter studies with larger, prospectively enrolled cohorts are needed to validate and refine this biomarker combination, confirm its clinical generalizability, and assess its performance in early-stage KD or incomplete presentations.

CRP, an acute inflammatory protein synthesized by hepatocytes in response to infection or tissue damage, plays a critical role in the body's innate immune response. It stimulates phagocytosis, the classical complement pathway, and pathogen and necrotic cell elimination by the immune system (39). Moreover, CRP also stimulates platelet aggregation, epithelial cell activation, and vascular permeability, which can cause vasculitis (40). Therefore, CRP's high serum levels suggest inflammation, making it a useful clinical diagnostic for myocardial injury severity and CAL prediction in acute KD. Our study indicated that KD patients had considerably higher CRP levels than non-KD febrile controls, suggesting its usefulness as a diagnostic tool.

MiRNAs, being small, stable molecules easily extractable from blood, urine, and other bodily fluids via cost-effective assays, have gained attention in KD biomarker research due to their high sensitivity, specificity, and stability. Over the last decade, numerous clinical studies have reported that dysregulated miRNAs may serve as potential KD indicators, utilizing a variety of methods, including qRT-PCR, miRNA microarray, next-generation sequencing, and bioinformatic analysis of Gene Expression Omnibus (GEO) database (41). However, no consensus miRNA-based KD diagnosis strategy has been widely applied in clinical practice.

Our findings on the upregulation of specific miRNAs in KD patients, including miR-222-3p, are consistent with previous reports. MiR-222-3p has been found significantly elevated in serum, platelets, and vascular endothelial cells in acute KD cases, playing a key role in vascular injury associated with the disease (42). This miRNA is shown to be predominantly involved in immune-related signalling pathways, notably affecting T cell and B cell receptor pathways, as shown by a recent KEGG pathway analysis. Research indicates miR-223-3p has a protective effect against vascular endothelial damage in KD (43). Studies by Wang et al. and Guo et al. highlight miR-223-3p's capacity to mitigate inflammation and attenuate endothelial cell injury by targeting and suppressing IL6ST, pSTAT3, NF-kB p65, and ICAM-1 in TNF-α treated human coronary artery endothelial cells (HCAECs) (44). Additionally, miR-223-3p influences cell proliferation, apoptosis, and inflammatory cytokine expression in HCAECs, partly by inhibiting FOXP3 expression. Its reduced levels have been linked to severe coronary artery lesions in KD through the modulation of vascular smooth muscle cell (VSMC) differentiation via the suppression of platelet-derived growth factor receptor b (PDGFRb) (45).

While miR-223-3p has been extensively studied, miR-19a-3p and miR-18a-5p have been less investigated in KD. Recent evidence indicates miR-19a-3p is upregulated in KD compared to non-KD febrile controls, however, researchers found it ineffective for predicting CAL or IVIG treatment responses. Subsequently, Liao et al. have identified a potential mechanism by which miR-19a-3p overexpression leads to endothelial dysfunction in KD. They demonstrated that miR-19a-3p suppresses Argonaute 2 (AGO2), disrupting the AGO2/PTEN/VEGF regulator*y* axis. This finding offers insight into the molecular pathways affected by miR-19a-3p in KD, highlighting its contribution to endothelial dysfunction. MiR-19a serve as a promising biomarker in heart and is essential for maintaining tissue homeostasis. Its increased levels have been associated with enhanced proliferation and reduced apoptosis in cardiomyocytes, contributing to protective effects against ischemia-induced heart failure in animal models and mitigating hypertension-induced cardiac hypertrophy by targeting PDE5A (46). Notably, miR-19a-3p also directly inhibits tumour necrosis factor alpha (TNF-α) (47), a pro-inflammatory cytokine linked to coronary artery abnormalities and is elevated in KD patients. MiR-18a-5p is also shown to be upregulated in KD, and its potential involvement in miRNA-18a-5p/Samd3 signaling pathway regulates endothelial injury, a key feature of KD pathophysiology. Beyond KD, miR-18a-5p has been shown to inhibit endothelial-mesenchymal transition through modulating the Notch2 pathway in cardiac fibrosis. Additionally, its elevated levels have been observed in atherosclerosis, indicating a broader impact on cardiovascular health (48). Furthermore, miR-18a-5p promotes the proliferation of vascular smooth muscle cells (VSMCs) by activating the AKT/ERK signaling pathway, highlighting its significant function in vascular pathology and potential as a biomarker across a spectrum of cardiovascular diseases.

Additionally, our study revealed a positive correlation among the expressions of miR-223-3p, miR-19a-3p, and miR-18a-5p, suggesting their involvement in shared biological pathways (Figure 3). We further attempted to illustrate the pathophysiologic role of these identified serum miRNAs in KD using bioinformatic tool miRNet 2.0, which serves as a powerful and convenient tool for target identification and visualisation. we identified 14 gene targets common to these miRNAs, including F3, TWF1, TP53, and others (Figure 4A). A further KEGG functional enrichment analysis highlighted their significant enrichment in pathways such as the p53 signalling pathway and cell cycle, among others, with the p53 signalling pathway showing a notable association with KD pathophysiology, considering a *p*-value <0.05, the functions of interest for KD, and total hits in the gene targets. P53, also known as TP53 or tumour protein, is a well-studied tumour suppressor that plays a crucial role in maintaining cellular and genetic stability and regulating cancer formation (49). Recent research has reported that miRNAs are implicated in the p53/miRNA network, that miRNAs regulated by p53 may mediate cellular processes such as cell cycle progression, epithelial–mesenchymal transition, metabolism, cell survival and angiogenesis (50). For instance, research has demonstrated that miR-223-3p directly targets *p53* to suppress cell proliferation and migration in lung squamous cell carcinoma (51). MiR-19a-3p was also implicated in *p53* pathway in ameliorating age-related bone loss by altering *p53* and *p21* expression (52). Moreover, genome-wide miRNA expression analysis has found that miR-18a-5p was regulated by *p53* in neuroblastoma to promote apoptosis in neuroblastoma cell lines (53). In KD, the activation of P53 by 1,25-Dihydroxyvitamin D3 suggests its involvement in regulating T lymphocyte proliferation and modulating ERK1/2 signaling pathway (54). Our study suggests a key role for the *p53*/miRNA network in KD's pathogenesis, revealing an underexplored area that warrants additional study.

In addition to the 14 gene targets, our network analysis revealed a new lncRNA target, KCNQ1OT1 (Figure 4B). Long non-coding RNAs (lncRNAs), which are transcripts longer than 200 nucleotides, play crucial roles in regulating gene expression and signalling pathways, impacting tissue homeostasis and diseases, including those affecting the cardiovascular system relevant to KD complications. LncRNA KCNQ1OT1, an antisense non-coding RNA implicated in cardiac development, has been observed to increase in myocardial infarction patients (55). Its downregulation has been shown to mitigate myocardial ischemia, reperfusion injury, and cardiac hypertrophy post-acute myocardial infarction by influencing the miR-2054/AKT3 and miR-204-5p/LGALS3 pathways (56). Interestingly, the specific involvement of LncRNA KCNQ1OT1 in KD has yet to be extensively explored. Our findings suggest a potential interaction between miRNAs and LncRNA KCNQ1OT1 in KD's pathophysiology, presenting a novel avenue for research. Further experimental research are needed to understand this relationship and its effects on KD.

In recent years, bioinformatics tools have been integrated in search of KD biomarkers and pathogenesis pathways. Biomarkers evaluated by urine peptidome profiling and whole blood cell type-specific gene expression analysis were used to construct the first diagnostic algorithm to distinguish KD from febrile controls in 2011 (57). However, its application was limited by a small sample size and extensive processing time. Recently, machine learning methods have been employed in KD diagnosis. For instance, Tsai et al. has recently introduced a KD prediction model established with XGBoost, which used 5 markers (Pyuria, white blood cell counts in urine, ALT level, CRP level, and eosinophil percentage) originated from 74,641 children with fever, and demonstrated a sensitivity of 93% and a specificity of 97% (58). Furthermore, a dynamic nomogram, developed using LASSO and incorporating 33 biological parameters from routine blood tests, effectively distinguishes KD from sepsis-a condition with similar symptoms with KD but requiring very different treatment (59). Our investigation offers a promising diagnostic approach for KD, suggesting the potential for a robust, clinically applicable algorithm. Future studies with expanded sample sizes are essential to validate our findings and refine this strategy. A more comprehensive and dynamic model, validated through extensive research, could significantly enhance KD diagnosis in clinical settings, contributing to timely and precise treatment interventions.

While this study focused on patients with ≥5 days of fever to align with diagnostic thresholds for KD, assessing the utility of this biomarker combination during the earlier febrile phase (<5 days) may enhance the timeliness of diagnosis and intervention. Future investigations should aim to evaluate the discriminative performance of CRP and miRNAs in this critical early window. Moreover, although febrile controls were used to simulate real-world diagnostic dilemmas, incorporating a healthy pediatric cohort would help establish baseline expression levels for these miRNAs and further clarify disease-specific alterations.

## Limitations

This study has several limitations. First, it was designed as a single-center, retrospective case-control study with a predetermined diagnosis. While this design allowed for timely sample collection and focused biomarker analysis, it is inherently different from a cohort study and does not permit longitudinal assessment of disease development or outcomes. Second, the relatively small sample size—particularly the limited number of complete KD case—may reduce the statistical robustness of the diagnostic model and raise concerns about potential overfitting. Although the model demonstrated excellent discrimination in this dataset, the findings should be considered exploratory and interpreted with caution. Validation in larger, independent cohorts is necessary to confirm the model's performance and ensure generalizability. Third, the study population consisted exclusively of Chinese pediatric patients, limiting extrapolation of the results to other ethnic groups. Future studies should aim to include diverse populations to assess the consistency of biomarker expression and model performance across different genetic and environmental backgrounds. Lastly, biomarker assessment was conducted at a single time point before IVIG administration. Evaluating longitudinal changes in CRP and miRNA expression—including in IVIG responders vs. non-responders—would further clarify their diagnostic and prognostic potential.

## Conclusions

This study presents a preliminary diagnostic approach combining CRP and serum miRNAs to distinguish KD from other febrile conditions. The integrated model demonstrated strong discriminative potential in this initial dataset and may offer a complementary strategy to enhance diagnostic confidence in clinically ambiguous cases. In addition, the involvement of these miRNAs in pathways such as p53 signaling and lncRNA interaction suggests possible mechanistic links that warrant further exploration. While the findings are promising, future studies with larger, diverse cohorts and prospective validation are essential before clinical implementation.

## Data Availability

The original contributions presented in the study are included in the article/Supplementary Material, further inquiries can be directed to the corresponding author.

## Supplementary material

Supplementary Table S1 Gene targets identified to be interacting with all three candidate miRNAs (miR-223-3p, miR-19a-3p and miR-18a-5p) by miRnet 2.0.

Supplementary Table S2 LncRNAs identified to be interacting with all three candidate miRNAs (miR-223-3p, miR-19a-3p and miR-18a-5p) by miRnet 2.0.

Supplementary Table S3 KEGG pathways implicated in candidate miRNAs and associated genes analyzed by miRnet 2.0.

Supplementary Table S4 KEGG pathways encompass all three miRNAs analyzed by miRnet 2.0.
